# SNP Selection in Genome-Wide and Candidate Gene Studies via Penalized Logistic Regression

**DOI:** 10.1002/gepi.20543

**Published:** 2010-11-18

**Authors:** Kristin L Ayers, Heather J Cordell

**Affiliations:** Institute of Human Genetics, Central ParkwayNewcastle upon Tyne, United Kingdom

**Keywords:** penalized likelihood, Lasso, elastic net, association analysis

## Abstract

Penalized regression methods offer an attractive alternative to single marker testing in genetic association analysis. Penalized regression methods shrink down to zero the coefficient of markers that have little apparent effect on the trait of interest, resulting in a parsimonious subset of what we hope are true pertinent predictors. Here we explore the performance of penalization in selecting SNPs as predictors in genetic association studies. The strength of the penalty can be chosen either to select a good predictive model (via methods such as computationally expensive cross validation), through maximum likelihood-based model selection criterion (such as the BIC), or to select a model that controls for type I error, as done here. We have investigated the performance of several penalized logistic regression approaches, simulating data under a variety of disease locus effect size and linkage disequilibrium patterns. We compared several penalties, including the elastic net, ridge, Lasso, MCP and the normal-exponential-γ shrinkage prior implemented in the *hyperlasso* software, to standard single locus analysis and simple forward stepwise regression. We examined how markers enter the model as penalties and *P*-value thresholds are varied, and report the sensitivity and specificity of each of the methods. Results show that penalized methods outperform single marker analysis, with the main difference being that penalized methods allow the simultaneous inclusion of a number of markers, and generally do not allow correlated variables to enter the model, producing a sparse model in which most of the identified explanatory markers are accounted for. *Genet. Epidemiol*. 34:879–891, 2010. © 2010 Wiley-Liss, Inc.

## INTRODUCTION

Regression methods are commonly used in statistical analysis, and the recent move to single marker testing in genetic association studies [WTCCC, 2007] has been out of necessity on account of the large number of predictor variables (upwards of 500,000 genetic markers) to be examined. Analyzing markers together in a regression model allows one to consider the impact of markers on other markers; a weak effect may become more visible when other causal effects are already accounted for, and a false signal maybe removed by the inclusion of a stronger signal from a true causal association. However, when the number of markers is larger than the number of test subjects or when variables are highly correlated, standard regression methods become overwhelmed. Penalized regression methods offer an attractive alternative. These methods operate by shrinking the size of the coefficients, pushing the coefficients of markers with little or no apparent effect on a trait down toward zero, reducing the effective degrees of freedom and in many cases performing model selection. Some penalization methods simply reduce the magnitude of the regression coefficients, while others coerce them to be zero. In genetic association analysis, we expect only a few markers to have a real effect on our trait (i.e. to be genuinely causal, or in linkage disequilibrium (LD) with a causal variant). Thus, through use of penalization, we can find the subset of markers most associated with the disease. One potential problem with penalization approaches is that a variable typically enters the model only if it significantly improves prediction. Thus, a variable with a strong marginal effect can be overlooked if other variables explain the effect. However, arguably, one would hope that the selection procedure would select the variables that do indeed best explain the data. Ideally, one strives to mimic the true underlying model, penalizing and thus eliminating non-causal loci, while leaving true causal loci unpenalized. A good penalty should result in minimally biased estimators, a sparse model and continuity to avoid instability in model prediction.

**Fig. 1:**
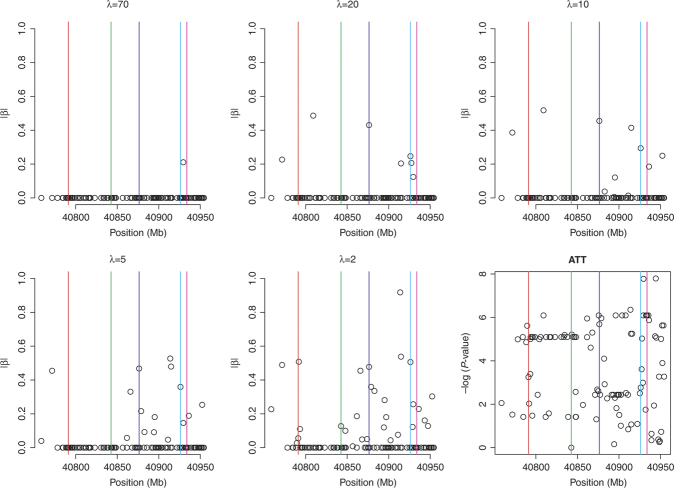
Analysis of simulated data from the *CYP2D6* gene region assuming five causal loci with MAFs <10%. The first five plots show the absolute values of the regression coefficients for the program *hyperlasso* [Hoggart et al., 2008] as the penalty parameter λ is relaxed. The final plot is the −log *P*-values for the Armitage Trend test. Each causal locus is marked by a vertical line.

As an illustrative example, see Figure 1. This figure shows association test results for simulated data in a region of high LD containing multiple causal loci. The trend test gives small *P*-values at many loci correlated with a causal locus, making it difficult to localize the causal locus. Penalized methods are more particular, selecting only one or several variables per causal locus.

In the genetics literature, use of these kinds of approach is just starting to emerge. Ridge regression has been used for distinguishing between causative and noncausative variants for quantitative traits [Malo et al., 2008]. For binary traits, the normal exponential-γ (NEG) distribution [Hoggart et al., 2008], elastic net [Cho et al., 2009] and group lasso [Croiseau and Cordell, 2009] have been applied in order to identify important individual single nucleotide polymorphisms (SNPs), while penalized logistic [Park and Casella, 2008] and least angle [Zhang et al., 2008] regression have been used for identifying gene-gene interactions.

Unfortunately, all penalization methods require specification of a penalization parameter (often referred to as the tuning or regularization parameter), and the parameter value yielding the optimal model is data driven. The choice of penalty parameter controls the number of variables selected: the larger the penalty, the smaller the selected subset. The value of the penalization parameter must be chosen, e.g. through cross validation, to avoid selection of a sub-optimal value. An additional problem with penalization approaches is that there are no efficient ways of obtaining a confidence interval or *P*-value for a coefficient. This must be done through a procedure such as bootstrapping, and still does not reflect a true *P*-value in the usual sense due to the complex selection procedure for the reduced model. Wu et al. [2009] have suggested calculating a leave-one-out index for each coefficient (based on likelihood ratio tests, leaving one variable out of the final model at a time), as a useful tool for comparing variables in the model. Another downfall of penalization is that many penalties not only shrink small coefficients but tend to overshrink what should be large coefficients, adversely affecting the value of the model in prediction. However, if the primary interest lies in locating possible disease genes, prediction per se is not of great concern.

Given the current interest in these types of method, the objective of this study is to evaluate the performance of several software packages for performing penalized logistic regression, using computer simulations. We focus on two different problems: first the issue of *detection* of a relevant SNP or region, and second the issue of *differentiation/localization* i.e. fine mapping within a region and distinguishing those variants that best explain the association and are thus most likely to be causal, or be in strongest LD with the causal variants. These two separate questions have not always been clearly distinguished in the literature—for example, Malo et al. [2008] compared ridge regression with simple and multiple linear regression using receiver operating characteristic (ROC) curves, without acknowledging that the hypotheses being tested by the different methods differed. (The ROC curves for simple linear regression counted detection of a locus in LD with the true functional variant as a type 1 error—as would be correct if one were using linear regression to try to differentiate between causal and noncausal variants—but in fact simple linear regression would generally be used for *detection* of effects, not for *differentiation* of effects, and so such an observation should actually have counted towards the power.)

We simulate data under a variety of disease locus effect size and linkage disequilibrium patterns. We compare several penalties, including the elastic net, the Lasso, a pseudo-ridge, the minimax concave penalty (MCP) and the NEG shrinkage prior to standard single locus analysis (the Armitage Trend Test) and simple forward stepwise regression. Software packages were selected for ease of use and to cover a wide range of penalty functions. We explore how markers enter a model as penalties or *P*-values are varied, and report the sensitivity and specificity for each of the methods.

## METHODS

Binary traits such as case/control status are generally analyzed using logistic regression. Given a dichotomous phenotype vector *Y* of *n* observations, and a matrix of SNP genotypes *X*, let *p* = *P*(*Y* = 1∥*X* = *x*). The likelihood is:


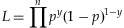


where


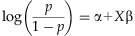


or equivalently,


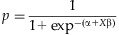


and β is our vector of coefficients.

In genetics, the advent of large-scale genotyping has lead to underdetermined problems where the number of markers is much larger than the number of individuals. In this case, standard logistic regression cannot produce a unique interpretable model. Penalized likelihood methods maximize the loglikelihood subject to a penalty which is dependent on the magnitude of the estimated parameters. A penalty on the likelihood will penalize models which have a large number of large regression coefficients, and thus will be optimized with a sparser model. In genetics, we have typically have many variables, but suspect that there are only a few underlying causal variants. An ideal penalty would quickly weed out variables with little effect, with only the most relevant variables remaining in the model. Use of a selection criterion, such as the Bayesian Information Criterion (BIC), is a form of regularization with a penalty that relies only on the number of coefficients, but not on their magnitude.

The most well-known penalty, *L*_1_ penalty, was introduced in the form of the least absolute shrinkage and selection operator or Lasso constraint by Tibshirani in 1996. The selection process in the Lasso is based on constructing continuous trajectories of regression coefficients as functions of the penalty level, which results in a more stable solution than subset selection methods [Efron et al., 2004]. To solve the Lasso regression maximization problem, methods such as least angle regression (LARS) [Efron et al., 2004], which can compute the entire piecewise linear path of the Lasso estimates for all values of the penalty parameters, or cyclic coordinate descent [Friedman et al., 2007; Wu and Lange, 2008] have been used. Lasso logistic regression is implemented in R packages such as *penalized* [Goeman, 2009], *glmnet* [Friedman et al., 2010], *grplasso* [Meier et al., 2008] and in other packages such as Bayesian binary regression (BBR) [Genkin et al., 2007] and the genetics software package Mendel [Wu et al., 2009]. The coefficient estimates from the Lasso procedure can also be interpreted in a Bayesian framework as posterior mode estimates using a Laplace (double exponential) prior on the coefficients [Tibshirani, 1996]. This observation has provided motivation for fully Bayesian approaches such as the Bayesian Lasso [Park and Hastie, 2008].

**Fig. 2:**
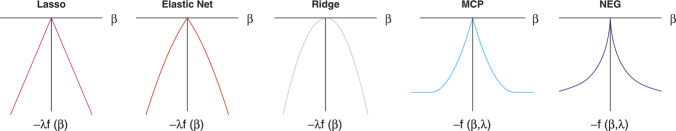
Plots of the negative of the penalty functions −λ*f*(β). The penalty (*y*-axis) is plotted against β (*x*-axis) for the Lasso, elastic net, ridge and MCP. The last plot is the NEG penalty *f*(β, λ), the log density of the NEG prior. The peaks of each function are at β = 0. In these plots, for each method, a λ value was selected to allow the penalty functions to be plotted on approximately the same scale. Other parameter values (such as the mixing parameter α in the elastic net) were set to the values used in the analysis.

The Lasso will encourage sparsity, setting most small coefficients to zero, due to the penalty function's sharp peak at zero. However, given a sufficiently large penalty parameter, the Lasso will also impose heavy shrinkage on large coefficients due to the absence of tails (constant rate of penalization), leading to biased coefficient estimates (see Fig. 2). A similar issue of bias is seen for other commonly used penalty functions such as the ridge (*L*_2_ penalty) [Hoerl and Kennard, 1970; Le Cessie and van Houwelingen, 1992], the elastic net (a mix of *L*_1_ and *L*_2_ penalties) [Zou and Hastie, 2005] and convex bridge regression (*L*_*q*_ penalty for 1<*q*<2). Many recent methods strive to relieve some of this bias by introducing penalties with flatter tails, so that large coefficients are only minimally shrunk (see Fig. 2). These include nonconvex bridge regression (*L*_*q*_ penalty for 0<*q*<1), the NEG prior implemented in *hyperlasso* [Hoggart et al., 2008] and thresholding penalties such as the smoothly clipped absolute deviation penalty (SCAD) [Fan and Li, 2001] and the MCP [Breheny and Huang, 2008; Zhang, 2007]. For a review of some of these methods and their properties, see Hesterberg et al. [2008].

In penalized likelihood inference, our objective function may be written as:


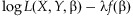


where the penalty *f* is a function of the regression coefficients (and possibly a mixing parameter). The amount of shrinkage is directly controlled by the derivative of the penalty function, and many different penalty functions have been proposed. For example, the elastic net criterion is:


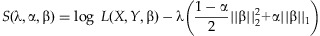


where 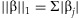 are the *L*_1_ and *L*_2_ norm, respectively. Here, λ controls the strength of the penalty while α is a mixing parameter that determines the strength of the *L*_1_ versus the *L*_2_ norm. The elastic net criterion above is reduced to the Lasso if we let α = 1. If α = 0, we have the *L*_2_ penalty used in ridge regression. The Lasso has been shown to be consistent for model selection under certain conditions given that the correlation between relevant (true) and irrelevant predictors is not too large [Zhao and Yu, 2006], or in other words, the selected model will contain the true model with high probability. However, the Lasso can select at most *n* (the number of observations) nonzero parameters, and cannot have a unique solution if any two variables are completely collinear. The elastic net is a stabilized version of the Lasso; it encourages groups of correlated variables to enter the model together, and thus a small change in the data will not have a large effect on the model. In our investigation, we use the *glmnet* software by Zou and Hastie [2005], to implement the Lasso, the elastic net with α = 0.4, and approximate ridge regression with α = 0.05. (We use approximate ridge regression because standard ridge regression only shrinks coefficients and does not set them to zero [Tibshirani, 1996], thus it does not automatically perform subset selection and one must compute a score for each coefficient for model selection.)

The MCP [Breheny and Huang, 2008; Zhang, 2007] is a nonconvex penalty that applies the same rate of penalization as the Lasso when the coefficients are near zero. The rate of penalization is the derivative or slope of the penalty function. As a coefficient moves away from zero, the rate of penalization is continuously relaxed until a defined threshold where the rate of penalization drops to zero. All coefficient values above this threshold contribute equally to the total penalty, so that very large coefficients do not increase the penalty too much, leading to less biased estimates of the large coefficients. The MCP is implemented in the R software package *grpreg*, and for our analyses we set *a* = 30, the default (where *a* is a tuning parameter related to the threshold at which the rate of penalization drops).

The method implemented in *hyperlasso* [Hoggart et al., 2008] is a Bayesian-inspired penalized maximum likelihood approach using a NEG prior. The NEG prior is a continuous prior distribution with a sharp mode at zero which has the effect of shrinking the regression coefficients heavily when they are near zero. The penalty function is derived by taking the logarithm of the prior, yielding a penalty that is a function of the coefficients squared and the logarithm of a parabolic cylinder function of the absolute value of the coefficients. For our analyses we set the shape parameter to 0.1.

All penalized regression methods require input of one or more values for the penalization parameter(s). From here on we will refer to this parameter as λ. The parameter value must be selected (e.g. through cross validation) to avoid selection of a sub-optimal parameter value. In general, a very large value of λ will only allow a small number of variables to enter the model. If we choose the value too small, the number of variables in the model may be too large, and our coefficients become less reliable because of their high variances; we approach standard regression with most of the variables included the model and overfitting occurs. The best choice of λ is data dependent and may vary, for example, from chromosome to chromosome, or window to window, within the same data set.

Due to the difficulties in finding an optimal value for λ, we found it most insightful to look at how variables enter a model as the penalization parameter is relaxed, or in nonpenalized methods as the *P*-value threshold for declaring significance is relaxed. Penalized methods tend to select only one or few variables belonging to a group of correlated variables, resulting in a sparse model. The question remains: are they missing anything? In our investigation we vary the value of λ to allow approximately only one additional variable enter the model at each step. We record the number of true positives and false positives at each value of λ for each method, along with the maximum LD between a false signal and a causal locus and the maximum LD between a missed causal locus and a signal.

We used computer simulation to compare five penalized methods (Lasso, elastic net (EN), ridge, MCP and NEG) to the Armitage trend test (ATT) and simple forward stepwise regression (FSTEP). All methods were run under the assumption of an additive allelic disease model. For the ATT, we look at how variables become significant as we increase the *P*-value threshold for declaring significance. In FSTEP, at each step the variable with the smallest *P*-value is added to the model, until no more variables reach the required threshold. We compare methods first with respect to *detection* of effects, in which detection of an allele in LD (*r*^2^>0.05) with a true causal variant counts as a success (and any other detection counts as a false positive), and second with respect to *localization/differentiation*, in which we only count detection of the true causal locus itself as a success.

### SIMULATION STUDY 1: DETECTION OF EFFECTS

Following the approach of Hoggart et al. [2008], we used the program FREGENE [Chadeau-Hyam et al., 2008; Hoggart et al., 2007] to simulate a population of 10,000 individuals with a sequence length of 20 Mb using recombination and mutation rates representative of a human population. We then thinned the data down to 4,000 SNPs with frequencies >0.01, retaining approximately one SNP every 5 kb. We then used the program SAMPLE [Chadeau-Hyam et al., 2008; Hoggart et al., 2007] to simulate 500 replicate data sets, requesting 1,000 cases and 1,000 controls generated from a model with six causal loci of varying allele frequencies of approximately 15, 5 and 2%, and risk ratios ranging from 1.4 to 3.0.

Genotype variables were used as predictor variables in each of the methods. Both *glmnet* and *grpreg* standardize the genotypes to have mean zero and variance 1 by default, and thus for comparability we also standardized the genotypes for the NEG and FSTEP. As the causal loci are not necessarily in the thinned SNP set, and our markers have low enough density that LD between them is fairly small, we report a true positive detection when a SNP of *r*^2^>0.05 with a causal variant is selected. A False-positive occurred when a SNP of *r*^2^≤0.05 was selected; however, a false positive was only recorded if it was more than 20 kb from a previously detected false positive, as was done by Hoggart et al. [2008]. This allows us to count a set of signals from a group of highly correlated variables as a single signal to prevent inflated false-positive rates in methods such as the ATT and the EN.

### SIMULATION STUDY 2: FINE MAPPING AND DIFFERENTIATION/LOCALIZATION OF EFFECTS

**Fig. 3:**
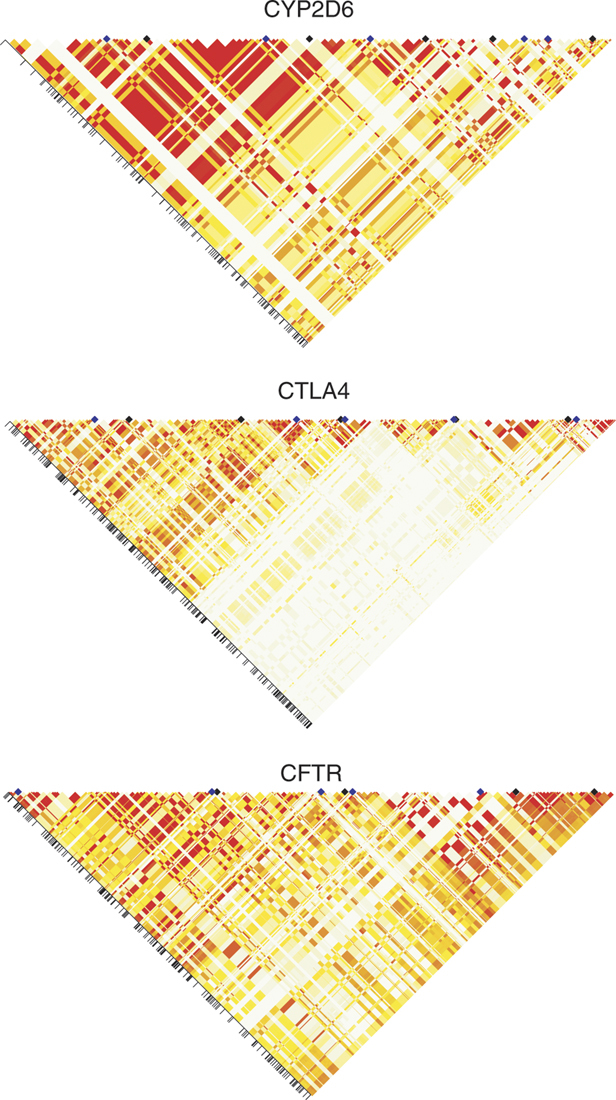
LD plots (pairwise *r*^2^) in three gene regions.

We also performed simulations based on three known gene regions of varying LD patterns (see Fig. 3): *CYP2D6*, *CFTR* and *CTLA4* containing 110, 190 and 228 SNPs, respectively. Although it would be possible to do standard logistic regression in this case (owing to the smaller number of markers compared to our previous simulation), the fact that the genotype variables are highly correlated leads to estimates with large variances.

With the software HAPGEN [Marchini et al., 2007], we simulated 500 replicate data sets for each of the three regions. We used the 120 haplotypes from the HapMap [The International Hapmap Consortium, 2003] CEU population as the basis of our simulations. In each replicate, a population of 20,000 individuals was created using the HapMap CEU haplotypes, and 1,000 cases and 1,000 controls were sampled from that population. For each region we considered six different underlying genetic model scenarios. We picked five “common” causal loci of varying but high minor allele frequency (see Table I) within each gene region. In scenarios 1–5, each locus in turn was used as the single (only) disease-causing variant, whereas in scenario 6, all five loci were assumed to act together multiplicatively to increase disease risk. The relative heterozygote and homozygote risk ratios in each scenario were set at 1.3 and 1.7, and the penetrance was chosen to give a population prevalence of approximately 10–13%. To compare the situation of common alleles having small effects with that of rare alleles having strong effects, the simulation was repeated using smaller minor allele frequencies, and relative risks chosen to give approximately 80% power for the detection of the individual SNP (see Table I).

**Table I:** Generating allele frequencies of the five causal loci

	Allele frequencies					
	
	RR of 1.3 and 1.7			RR to give 80% power		
		
	Gene			Gene		
		
Locus	CYP2D6	CFTR	CTLA4	CYP2D6	CFTR	CTLA4
1	0.325	0.050	0.375	0.100	0.050	0.030
2	0.050	0.175	0.450	0.008	0.080	0.008
3	0.192	0.475	0.183	0.025	0.025	0.050
4	0.242	0.242	0.275	0.043	0.008	0.025
5	0.450	0.367	0.050	0.017	0.042	0.017

The first set correspond to common alleles and were used with heterozygote/homozygote relative risks set at 1.3 and 1.7. The second set correspond to rare alleles with relative risks chosen to give approximately 80% power.

The simulated data were analyzed using the same five penalized methods as in Simulation Study 1. Since in this second experiment we were interested in the performance of the methods with respect to differentiation of SNPs, in common with Malo et al. [2008] we counted detection as selection of the true causal locus and considered all other detections a false positive. However, in a similar manner to Simulation 1, we considered a false signal in very close proximity (10 kb) to a previously reported false positive as a single false-positive signal.

## RESULTS

### SIMULATION STUDY 1: DETECTION OF EFFECTS

**Fig. 4:**
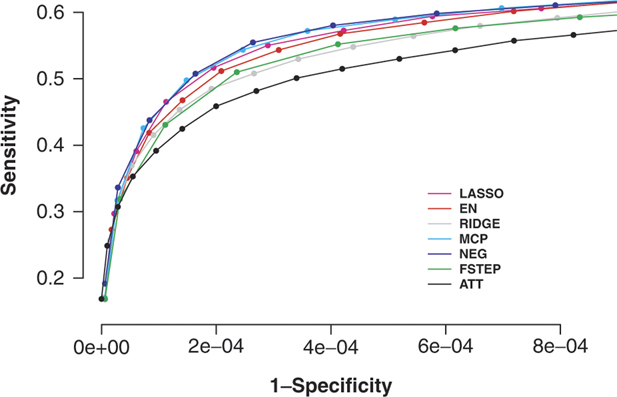
Sensitivity (detection rates) versus 1-specificity (false-positive rates) as the penalty parameter λ is varied. Results are for seven different methods over 4,000 simulated SNPs with six causal loci. Note the difference in axis scales, as we are interested in low false-positive rates.

With respect to detection, Figure 4 shows that the penalized approaches all outperform the ATT and FSTEP, and NEG slightly outperforms the other methods. We might expect penalties with flatter tails to perform better as they are less likely to allow important variables to leave a model after they have entered. The Lasso appears to pull the more weakly related parameters to zero faster than the elastic net or ridge regression does. Thus, penalties that do not have a strong peak at zero tend to let in too many weakly correlated variables leading to larger amounts of false positives. Forward stepwise regression performs slightly worse than the penalized methods, probably due to the fact that it is greedy, making poorer decisions in variable selection as the model grows larger.

### CHOICE OF λ

Figure 4 shows the relationship between true and false-positive detection for each of the methods as λ is varied. However, there is always the question of which penalization parameter value to use in practice (i.e. which value of λ is optimal). Some software uses either cross validation with training and testing data sets, or bootstrapping, both of which can be computationally intensive. Cross-validation uses prediction accuracy to find the best penalty, which can lead to models that contain too many false positives, i.e. variables which are not true predictors [James and Radchenko, 2009; James et al., 2009]. Selection criteria such as the Aikike Information Criteria (AIC), BIC and generalized cross validation (GCV) have been suggested. However, in genome-wide association studies, we are less interested in prediction performance than we are in identifying important predictors (while not identifying too many false positives). If we desire to control the false-positive rate, say at 1 in 100,000 (10^−5^), one way to get a rough estimate of the appropriate λ is to use permutation. For Simulation Study I above, to calculate the appropriate value of λ in each replicate, we did 25 permutations of the case/control status and recorded the value of λ at which the first variable entered the model in each permutation. Since in each simulation replicate there are 4,000 markers and 25 permutations (for a total of 100,000 tests), we choose the largest value of the recorded λ from the 25 permutation replicates as an estimate of the λ that allows only one variable out of 100,000 markers to enter the model for that simulation replicate. (In a study with 100,000 markers genotyped, one could instead perform a single permutation and choose the value of λ that allows a single marker to enter the model.) Table II demonstrates that the value of λ chosen in this way was reasonable, giving a false-positive rate of the correct order of magnitude. In the case of a large number of markers, this process is also much faster than doing cross validation, as it is not necessary to run a large number of different values for λ. The *P*-value cut off for the FSTEP and ATT (which do not require specification of a penalization parameter) for this comparison was set at 10^−5^, which similarly produced a false-positive rate of the correct order of magnitude.

**Table II:** Simulation Study 1: true and false-positive counts and rates for best λ

	Method						
	
	Lasso	EN	RIDGE	MCP	NEG	FSTEP	ATT
No. of true detections	1,163	1,167	1,180	1,152	1,110	1,172	1,174
Detection rate	0.3877	0.3890	0.3933	0.3840	0.3700	0.3907	0.3913
No. of false positives	36	40	52	35	25	30	44
False-positive rate	1.80e − 05	2.00e − 05	2.60e − 05	1.75e − 05	1.25e − 05	1.50e − 05	2.20e − 05

The procedure described above estimates a (potentially different) value of λ in each simulation replicate. A more accurate estimate of λ could be obtained by using all 500 simulation replicates, taking advantage of the fact that there should be a single value of λ that gives the same desired expected type I error in each replicate. This approach, however, could not be implemented in a real study that consists of essentially a single data replicate. Hoggart et al. [2008] obtained an explicit expression for the approximate type-I error of the NEG, so that it can be calibrated without recourse to permutation techniques. However, this functionality is not currently implemented within their software. For the group lasso method, Meier et al. [2008] proposed using a fixed value of λ based on the number of groups of predictors being considered; however, for genetic studies, this choice of λ was found to work poorly in practice [Croiseau and Cordell, 2009].

### SIMULATION STUDY 2: FINE MAPPING AND DIFFERENTIATION/LOCALIZATION OF EFFECTS

**Fig. 5:**
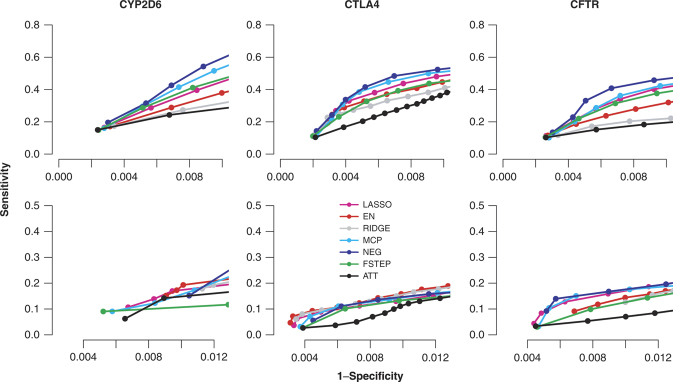
Sensitivity versus 1-specificity as the penalty parameter λ is varied in gene regions. The results for each gene region under scenario 6 (five causal loci). The top row shows results for rare causal alleles while the bottom row shows results for common alleles.

With respect to differentiation/localization, in common with previous studies [Hoggart et al., 2008], we found penalized methods to perform better than single marker tests (see Fig. 5). Single locus analysis was less able to differentiate between true causal SNPs and those correlated with the true causal SNPs. In the case of rare causal alleles, our results are similar to those in the Simulation Study 1, with the NEG slightly outperforming the other methods, and the ATT again performing most poorly. However, with extremely common alleles, all methods perform rather poorly, with forward stepwise regression and the ATT doing slightly worse than the other methods. We also tried using smaller relative risks but all of the methods were somewhat under powered, and the results were less interpretable, although there was the same general trend (data not shown). The common alleles tended to be in very high LD with many markers, and because we only recorded detection if we found the true causal locus, detection was quite difficult.

### CHOICE OF **λ**

We again compared the methods with respect to achieving a given error rate. Our desired false-positive rate was 0.05 divided by the number of markers, a Bonferroni correction. Within each replicate we therefore performed 20 permutations (1/20 = 0.05) and picked the maximal value of λ leading to just one marker entering the model, in order to obtain an estimate of the appropriate λ in the same way as before.

For both rare and common causal alleles (Tables III and IV), our false-positive rates for all methods are higher than we would expect. For example, in *CYP2D6*, we would hope for 0.05/110 = 0.00045. This inflation in false-positive rate may be due to the fact that we have simulated causal loci in regions of very strong LD. The inflation in false-positive rates was even higher with common causal alleles where the degree of LD between other markers and these loci was larger. There are many markers in the model that are in very strong LD with our causal locus but are counted as a false positive. The fact that the penalized methods again give the same order of magnitude of false-positive rates as the ATT test (which is based on a *P*-value cut off) is confirmation that using permutation to pick the best penalization parameter seems a reasonable thing to do. Comparing the methods, forward stepwise regression seems to be the most conservative method, but is least powerful, while the ATT has high error rates and reasonable power, but becomes less powerful than the other methods as the *P*-value threshold is relaxed. Of the penalized methods, the NEG is the most conservative, with very little loss of power over the other methods, while the ridge penalty allows far too many variables in the model.

**Table III:** Simulation Study 2: true and false positives for best λ for rare alleles with relative risks chosen to give approximately 80% power

	Method						
	
	Lasso	EN	RIDGE	MCP	NEG	FSTEP	ATT
CYP2D6							
No. of true detections	722	762	802	697	665	508	609
Detection rate	0.29	0.30	0.32	0.28	0.27	0.20	0.24
No. of false positives	232	335	681	220	187	151	574
False-positive rate	0.0044	0.0064	0.0130	0.0042	0.0036	0.0029	0.0109
CTLA4							
No. of true detections	442	472	539	444	417	332	432
Detection rate	0.18	0.19	0.22	0.18	0.17	0.13	0.17
No. of false positives	237	284	395	253	215	223	387
False-positive rate	0.0021	0.0026	0.0035	0.0023	0.0019	0.0020	0.0035
CFTR							
No. of true detections	418	432	469	380	374	256	242
Detection rate	0.17	0.17	0.19	0.15	0.15	0.10	0.10
No. of false positives	340	618	1297	313	261	218	823
False-positive rate	0.0037	0.0067	0.014	0.0034	0.0028	0.0024	0.0089
Average ratio of True Positives to False Positives							
	1.96	1.35	0.76	1.94	2.20	1.85	0.72

Results are given for Scenario 6 (five causal loci).

**IV:** Simulation Study 2: true and false positives for best λ for common alleles with relative risks of 1.3 and 1.7

	Method						
	Lasso	EN	RIDGE	MCP	NEG	FSTEP	ATT
CYP2D6							
No. of true detections	811	1,310	1,714	599	551	372	1,061
Detection rate	0.32	0.52	0.69	0.24	0.22	0.15	0.42
No. of false positives	1,480	3,881	6,819	652	586	702	4,700
False-positive rate	0.0282	0.0739	0.1299	0.0124	0.0112	0.0134	0.0895
CTLA4							
No. of true detections	435	687	970	367	307	286	723
Detection rate	0.17	0.27	0.39	0.15	0.12	0.11	0.29
No. of false positives	1,470	2,215	3,597	1,120	817	901	2,495
False-positive rate	0.0132	0.0199	0.0323	0.0100	0.0073	0.0081	0.0224
CFTR							
No. of true detections	503	972	1256	342	296	113	849
Detection rate	0.20	0.39	0.50	0.14	0.12	0.05	0.34
No. of false positives	1019	2293	4202	598	508	478	2500
False-positive rate	0.0110	0.0248	0.0454	0.0065	0.0055	0.0052	0.0270
Average ratio of True Positives to False Positives							
	0.44	0.35	0.27	0.55	0.60	0.37	0.27

Results are given for Scenario 6 (five causal loci).

For more common alleles, the results are less clear (Table IV). Even though the effect size is quite small, we should still have high power to detect common alleles. However, the high LD seems to detract from the ability to distinguish between true causal loci and those variants in LD with them. While rare alleles are not in LD with many markers, common alleles are in high LD with many, not necessarily adjacent, markers.

**Fig. 6:**
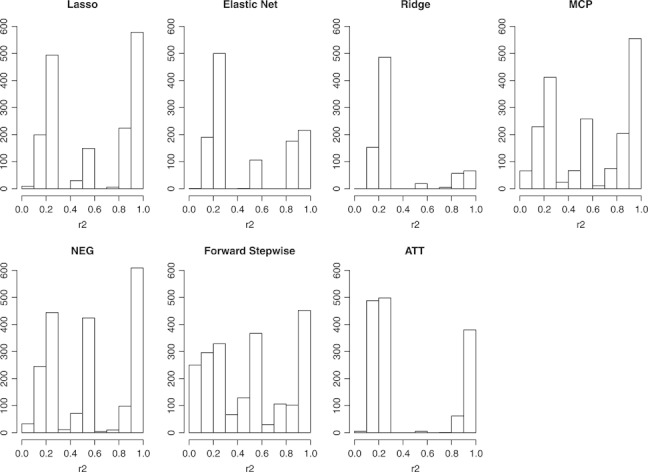
Maximum LD of missed causal loci with detected loci. Results are shown as histograms of maximum LD (*r*^2^) of a missed causal locus with markers in the model. Presented are the results for the *CYP2D6* gene region under scenario 6 (five common causal alleles) for the λ value chosen through permutation, where the *y*-axes are the counts over the 500 replicates.

**Fig. 7:**
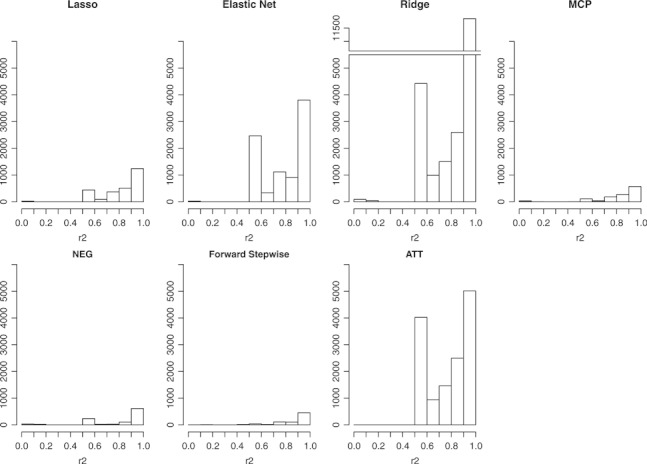
Maximum LD of false positives with causal loci. Results are shown as histograms of maximum LD (*r*^2^) that a false positive shares with any causal locus. Presented are the results for the *CYP2D6* gene region under scenario 6 (five common causal alleles) for the λ value chosen through permutation, where the y-axes are the counts over the 500 replicates.

On closer inspection of our results, we found a good proportion of the false positives in all methods to be in high LD with a true causal locus. In all methods but the ridge, and to a lesser extent the elastic net, if a true causal locus was missed, then there is often a marker in the model that is in high LD with the causal locus which gets selected instead of the true causal locus (see Figs. 6 and 7). To see if there were a large number of false positives perfectly correlated with a causal locus, we repeated the simulation for the common alleles in the *CYP2D6* region which had the highest level of LD. We did not count these perfectly correlated variables as false positives, and counted them as a detection if the causal locus was not originally detected. As expected, we found that the sensitivity improved, and the false-positive rate dropped, but that the effect was relatively small and fairly uniform across the different methods.

The above results relate to scenario 6, in which five causal variants were simulated within each gene region. We also performed the same comparison for scenarios 1–5, in which only one causal allele was simulated in each gene region (see Table V). With regards to a causal locus, the Lasso, NEG, forward stepwise regression and MCP often select only one of a group of highly correlated variables, and if the wrong one is selected, the true causal locus has little chance of entering the model at a later step, as is evident by flat sensitivity versus 1-specificity curves (data not shown). This may occur when the disease locus is in perfect LD with another markers or in any replicate where a marker in very high LD with the causal locus is more strongly associated with disease status than the causal locus by chance. For rare causal alleles, we found FSTEP performed best (with the highest ratio of true detections to false positives), with NEG coming in second place. For common causal alleles, there was less difference between the methods, but FSTEP and NEG again performed best in two out of the three gene regions (*CYP2D6* and *CTLA4*).

**V:** Ratio of detections over false positives for best λ for scenarios 1–5 (each involving a single causal locus)

	Rare alleles					Common alleles				
		
	Scenario					Scenario				
		
Gene and Method	1	2	3	4	5	1	2	3	4	5
CYP2D6										
Lasso	2.041	4.893	5.643	3.083	0.242	0.044	2.171	1.147	0.184	0.675
EN	1.589	4.029	5.097	2.921	0.273	0.102	1.902	0.648	0.306	0.597
RIDGE	0.764	1.557	3.160	1.606	0.250	0.115	0.876	0.203	0.205	0.254
MCP	1.743	2.600	2.937	1.654	0.166	0.039	1.160	1.082	0.090	0.766
NEG	3.022	5.952	5.920	3.062	0.089	0.009	2.033	1.409	0.027	0.560
FSTEP	3.964	20.333	20.333	7.273	0.286	0.036	4.556	1.387	0.098	0.907
ATT	0.663	1.547	6.611	1.878	0.245	0.144	1.467	0.196	0.157	0.171
CTLA4										
Lasso	2.310	1.076	0.358	4.325	3.794	0.204	0.234	0.192	3.491	0.157
EN	1.971	0.966	0.723	4.375	3.639	0.369	0.756	0.486	2.787	0.256
RIDGE	0.841	0.923	1.000	2.966	2.848	0.238	0.633	0.472	1.172	0.380
MCP	1.291	0.952	0.174	2.667	2.173	0.108	0.099	0.107	3.267	0.062
NEG	2.714	2.756	0.066	4.056	3.793	0.014	0.002	0.017	4.532	0.056
FSTEP	3.714	2.059	0.153	12.182	6.188	0.126	0.101	0.095	7.857	0.088
ATT	0.785	1.079	0.597	4.786	2.857	0.203	0.458	0.308	1.406	0.488
CFTR										
Lasso	1.211	0.530	6.400	4.906	0.532	0.833	1.568	0.238	0.143	0.202
EN	0.784	0.439	5.848	3.810	0.645	0.738	1.089	0.250	0.204	0.619
RIDGE	0.306	0.278	3.386	2.205	0.555	0.308	0.419	0.141	0.159	0.314
MCP	1.281	0.421	3.067	1.693	0.270	0.602	1.514	0.241	0.082	0.071
NEG	1.929	0.366	6.250	5.833	0.141	1.022	2.388	0.293	0.005	0.009
FSTEP	2.095	0.727	34.250	59.500	0.220	1.474	2.727	0.214	0.099	0.081
ATT	0.251	0.239	8.667	3.867	0.532	0.343	0.345	0.136	0.174	0.244

## DISCUSSION

In this study we have examined the performance of a variety of different penalized regression approaches and compared their performance with respect to (a) detection and (b) distinguishing of true from false causal variants in genetic association studies. Although the performance of some of the individual methods we considered has previously been examined, to our knowledge there has been no comprehensive comparison between methods and between such methods and the simpler approaches that are often used in genetic association studies (such as the Armitage trend test and forward stepwise regression). Moreover, some previous studies [Malo et al., 2008] have been plagued by confusion over whether the methods were being assessed with respect to (a) or (b).

Overall, we found broadly similar performance between the different penalization methods, with NEG giving the overall best and the ATT the overall worst performance. Although larger parameter estimates are always more heavily penalized, methods that apply larger relative penalties on small parameter estimates and relatively lower penalties to larger estimates seem to perform better and more accurately estimate the effect size of the selected SNPs. These penalties prevent variables that enter the model early from leaving the model later on, and exclude the entry of additional variables whose effects are solely due to correlation with already included variables. As we relax the error rate, penalized methods outperform the ATT in detection, and may be useful for further exploration of causal variants.

Although forward stepwise regression is conservative for very stringent *P*-values, for more relaxed *P*-values it generally performed slightly worse than the penalized methods with a higher false-positive rate and lower sensitivity, although it did perform well with respect to fine mapping in the simulations where only a single causal variant existed in a region. Forward stepwise regression has the disadvantage that once a variable enters the model it cannot correct itself by removing it if other variables are a better fit; it is known to be greedy and unstable [Breiman, 1996]. However, a forward/backward selection procedure might perform better, although penalization methods would still be expected to be less greedy. Penalized methods have previously been shown to give superior performance over stepwise elimination/addition algorithms that often lead to local rather than globally optimal solutions [Breiman, 1995]. However, in spite of their theoretical limitations [Hastie et al., 2001], stepwise regression approaches [Cordell and Clayton, 2002] have frequently been used to differentiate between potentially causal and noncausal variants in genetic association studies [Barratt et al., 2004; Plenge et al., 2007; Scott et al., 2007; Ueda et al., 2003] and appear to work rather well in practice [Charoen et al., 2007].

As well as giving the overall best performance in our study, the NEG has the advantage of being genuinely applicable to genome-wide data comprising many thousands of predictor variables, unlike most of the other penalized approaches we considered, which suffer from limitations with respect to the number of markers that can be considered simultaneously [Croiseau and Cordell, 2009]. Given the recent success of single-marker approaches in detecting effects in genome-wide association studies [Easton et al., 2007; Fellay et al., 2007; Frayling et al., 2007; Todd et al., 2007; WTCCC, 2007; Zeggini et al., 2008, 2007], in practice it is hard to imagine not undertaking a first-pass single-locus analysis. However, the superior performance of the NEG with respect to detection as well as with respect to differentiation/localization of effects suggests that, for most genome-wide studies, a follow-up analysis using the NEG or similar (to generate a sparser model in which the most important explanatory markers are accounted for) would be a worthwhile undertaking.
